# Magnetic Resonance Image-Guided Hypofractionated Ablative Radiation Therapy for Hepatocellular Carcinoma With Tumor Thrombus Extending to the Right Atrium

**DOI:** 10.7759/cureus.23981

**Published:** 2022-04-09

**Authors:** Neris Dincer, Gamze Ugurluer, Teuta Zoto Mustafayev, Gorkem Gungor, Banu Atalar, Koray Guven, Enis Ozyar

**Affiliations:** 1 Radiation Oncology, Acibadem University, Istanbul, TUR; 2 Radiation Oncology, Acibadem Maslak Hospital, Istanbul, TUR; 3 Radiology, Acibadem University, Istanbul, TUR; 4 Radiation Oncology, Acibadem Mehmet Ali Aydinlar University School of Medicine, Istanbul, TUR

**Keywords:** mr-linac, sbrt, tumor thrombus, inoperable, hepatocellular cancer

## Abstract

Hepatocellular carcinoma (HCC) presenting with tumor thrombus (TT) and inferior vena cava (IVC)/right atrium (RA) infringement point to an advanced-stage disease that is deemed inoperable. Stereotactic body radiotherapy is an emerging treatment option for this group of patients with promising outcomes in recent studies that are comparable to conventional treatment methods, namely, transarterial chemoembolization and transarterial radioembolization. Here, we report a case of HCC with RA extension through the IVC. The patient was referred to our clinic for treatment options, and he was found suitable for magnetic resonance imaging-guided radiotherapy (MRgRT). We treated the patient with MRgRT in five fractions to a total dose of 40 Gray. The tumor was tracked during the treatment sessions, and adaptive treatment planning was performed before each fraction. The patient tolerated the treatment well with no acute grade 3-4 toxicities. The last follow-up showed that the patient had a complete biochemical response and is now a candidate for an orthotopic liver transplant. To our knowledge, this report is the first to document the MRgRT treatment of an HCC with TT and RA extension. MRgRT is safe and feasible for this patient group and can be an effective bridging therapy for liver transplants.

## Introduction

Hepatocellular cancer (HCC) is the most common primary liver malignancy with an increased incidence over the last four decades [1]. Although the treatment of choice is surgery, fewer than 20% of patients are amenable to surgery at the time of presentation [2]. Until recently, transarterial chemoembolization (TACE) and transarterial radioembolization (TARE) were the preferred non-curative methods for patients unsuitable for surgery [3]. With the new technologies that enable administering high doses to the tumor with safer side effect profiles, radiotherapy (RT) can be a potential alternative to the abovementioned methods.

HCC presenting with tumor thrombus (TT) is considered inoperable and has an even worse prognosis. Studies reporting treatment of HCC with TT via surgical resection, TACE, and TARE have been reported in the literature [4]. Stereotactic body radiotherapy (SBRT) has previously been reported to be effective in HCC patients with TT in the portal vein [5,6]. In such cases, adaptive treatment under daily image guidance should be considered to cover the target volume. Computed tomography (CT) images do not allow precise identification of liver volumes, as well as daily in-place adaptive planning; thus, magnetic resonance imaging-guided radiotherapy (MRgRT) can be beneficial in HCC, especially in the presence of TT [7,8]. Here, we present a case of HCC with TT extending to the right atrium (RA) treated with MRgRT. To our knowledge, this is the first report to document the MRgRT treatment of an HCC with TT and RA extension.

## Case presentation

Our patient is a 64-year-old male. His medical history included thalassemia and diabetes mellitus. His family history was free of malignant diseases. In 2020, the patient presented to a tertiary care center with complaints of fatigue and pain in the right lumbar area. Serology revealed that the patient was positive for hepatitis C virus. The patient was diagnosed with cirrhosis with a Child-Pugh score of 6 and a Model for End-Stage Liver Disease (MELD) score of 12. Further workup was consistent with portal hypertension and esophageal varices secondary to cirrhosis. Alpha-fetoprotein (AFP) level was 658 ng/mL which was extremely above the normal reference range. CT was consistent with an inoperable HCC at segments three and four of the liver. Laparoscopic biopsy ensued CT, and the pathology report confirmed the diagnosis. In September 2020, the patient underwent TACE. CT was performed one month after the procedure and showed a residual mass of 18 × 14 mm at segment three, as well as a TT in the portal vein and lymph nodes in the celiac trunk and periportal area. The patient was found unsuitable for transplantation, and radioembolization was recommended for him. Although the liver function tests were slightly above the normal reference range, the patient had an increased bilirubin level, so radioembolization could not be performed. In January 2021, the patient consulted our clinic with his current CT and magnetic resonance imaging (MRI) scans. His general health was good, and he had no restrictions in performing his daily activities. His blood test results were significant for AFP level of 653 ng/mL and total bilirubin of 104 µmol/L. He had mild anemia (hemoglobin was 10.4 g/dL). His current radiological imaging showed HCC at segments three and four with a thrombus in the inferior vena cava (IVC). The patient was presented to a multidisciplinary tumor board for treatment options, namely, radioembolization and radiotherapy. Because radioembolization was found to be unsuitable for the patient, we decided that the patient was an eligible candidate for SBRT.

**Figure 1:**
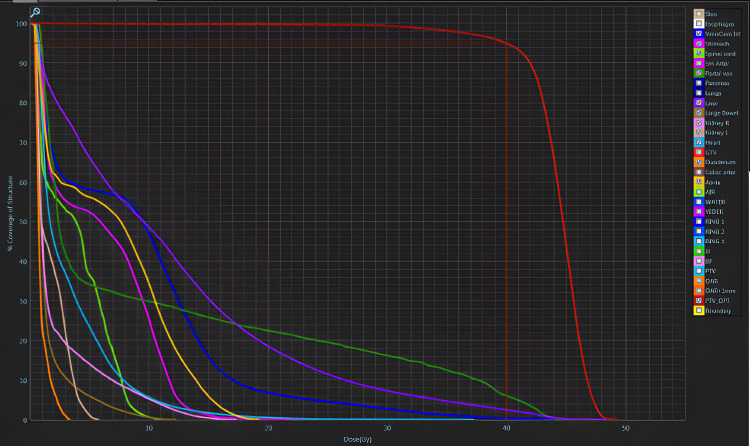
Dose-volume histogram of the reference plan. Red line: planning target volume; dark blue line: inferior vena cava; purple line: liver; green line: portal vein; brown line: large Bowels; orange line: duodenum; light blue line: heart; light green line: spinal cord; light pink line: right kidney; light brown line: left kidney

**Figure 2:**
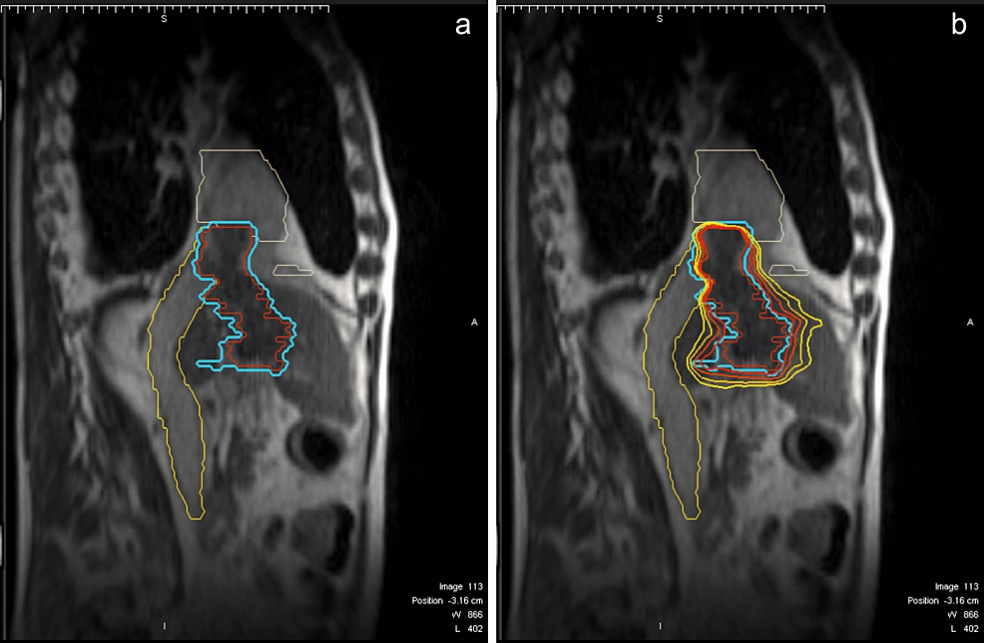
GTV, PTV, and isodose colorwash view in the sagittal plane. (a) GTV (red line) and PTV (blue line), tumor thrombus extending the atrium. (b) GTV, PTV, and isodose lines in the sagittal plane. The heart is contoured in pink, and the inferior vena cava is contoured in yellow. GTV: gross tumor volume; PTV: planning target volume

MRI-guided SBRT at the MRIdian Linac (ViewRay IncMountain View, CA, USA) was deemed suitable for this patient. The patient was put on a simulation through MRIdian Linac to ensure that the target volumes are clearly visible and distinguishable from the normal tissue in the MRIdian Linac System. The image was acquired in 25 seconds via TrueFISP sequence in three-dimensional 0.35 Tesla MRI with no contrast material injection as the tumor was visible in non-contrast images. Thereafter, the patient underwent simulation CT. Both simulations were performed with the deep-inspiration breath-hold (DIBH) technique. Pretreatment simulation MR Linac scans were fused with the simulation CT scans and other available diagnostic images. Gross tumor volume (GTV) was delineated as the primary liver lesion and the TT extending to the RA. The planning target volume (PTV) was extended 3 mm in all directions from the GTV. Organs at risk (OARs) were determined as the esophagus, stomach, spinal cord, superior mesenteric artery, portal vein, pancreas, lungs, liver, bowels, right and left kidney, heart, duodenum, celiac artery, and aorta. Volumes are presented in Table 1. A total dose of 40 Gray (Gy) in five fractions (8 Gy/fraction) was prescribed for the lesion. A step and shoot intensity-modulated radiotherapy (IMRT) treatment plan was generated according to departmental dosimetric dose constraints (Table 2) with both arms-down positions (patient was unable to lay in arms-up position for a whole treatment session) with 23 fields and one isocenter and 6 MV flattening filter-free (FFF) photons. Pixel size and dose grid resolution were 0.3 cm, and the number of multileaf collimators (MLC) was 92. IMRT efficiency was 8. V89 of PTV had 40 Gy and V95 of GTV had 39 Gy. This reference plan was denoted as “Plan 0.” Dose-volume histograms are shown in Figure 1, and Figure 2 shows the GTV, PTV, and isodose lines with the tumor extending to the IVC and heart.

**Table 1:** Volumes of structures in the reference plan (Plan 0). GTV: gross tumor volume; PTV: planning target volume

Structure	Volume (cc)
GTV	83.2
PTV	128.5
Liver	1833
Large bowel	219
Right kidney	207
Left kidney	171.2
Heart	554
Duodenum	77
Stomach	252

**Table 2:** Institutional constraints for OAR doses in MRgRT. OaR: organs at risk; MRgRT: magnetic resonance imaging-guided radiotherapy; Gy: Gray; cc: cubic centimeter; PTV: planning target volume; IVC: inferior vena cava; SMA: superior mesenteric artery

Structure	Maximum dose (Gy)	Dose to volume
PTV	N/A	>95% at 40 Gy
Duodenum	N/A	≤1 cc at 33 Gy
Duodenum	N/A	≤0.5 cc at 36 Gy
Large bowel	N/A	≤1 cc at 33 Gy
Large bowel	N/A	≤0.5 cc at 36 Gy
Stomach	N/A	≤1 cc at 33 Gy
Stomach	N/A	≤0.5 cc at 36 Gy
Spinal cord	25	≤10 cc at 25 Gy
Spinal cord	25	≤1.2 cc at 14.5 Gy
Spinal cord	25	≤0.35 cc at 23 Gy
Aorta	53	N/A
IVC	53	N/A
Esophagus	35	≤5 cc at 19.5 Gy
SMA	53	N/A
Portal vein	53	N/A
Liver	N/A	≤700 cc at 15 Gy
Right kidney	10	N/A
Left kidney	10	N/A
Heart	36	≤15 cc at 32 Gy

The patient was treated every other day. An adaptive online plan was generated while the patient was on the treatment table, and online quality assurance (QA) was obtained before each radiotherapy fraction. A radiation oncologist evaluated the daily anatomy and contoured the GTV, and adaptive planning was performed at the beginning of each fraction. An adaptive approach was applied in all fractions. Two plans were generated during each fraction; the baseline plan was recalculated on the anatomy of the day and a reoptimized plan which used the same number and direction as the baseline plan. The daily plan adaption is comprehensively described in our previous case report [9]. The reasons for reoptimized adaptive plans were inadequate PTV coverage and dose violation to the heart for the first, second, fourth, and fifth fractions and inadequate PTV coverage alone for the third fraction. The institutional constraints were used to evaluate the doses to OAR and target volume (Table 2).

The patient tolerated the treatment well without any acute grade 3-4 toxicity. He did not have any serious complaints including any pain but a feeling of swelling on the right side of the abdomen and mild fatigue. On the 16th day post-treatment, the patient expressed progression of the swelling and pain sensation. The patient then went back to his hometown and sent us his laboratory results which revealed a tendency of decline in AFP levels that were 24 ng/mL, 6.84 ng/mL, and 3.77 ng/mL in the first, second, and fourth months, respectively, after the RT. Eight months after the treatment, the patient was free of symptoms and applied for orthotopic liver transplantation. The AFP level was 2.80 ng/mL. Cardiovascular, psychiatric, and respiratory medicine examinations were performed and the patient was found suitable for the operation. The abdominal CT with contrast showed regression in the treated lesion which was confirmed by the positron emission tomography-computed tomography scan. CT angiography revealed a 10 mm diameter thrombotic filling defect not reaching the RA which is also consistent with regression. The patient will undergo orthotopic liver transplantation.

## Discussion

HCC is one of the most common cancers causing significant morbidity and mortality. Although liver transplantation is the mainstay of treatment, fewer than 20% of patients are amenable to surgery at the time of diagnosis [2]. Treatment modalities such as TACE and TARE are preferred as bridging therapies for transplantation or for local control in these patients [3].

The presence of TT constitutes a contraindication for surgery due to the risk of tumor spread in HCC patients. This patient group also has a high recurrence risk [10]. TACE can be safely used if the remaining liver tissue function is good and the collateral blood flow is sufficient, otherwise it poses the risk of ischemic necrosis [11]. TARE is also effective and well tolerated but it works well with patients with good basal prognostic factors such as non-metastatic HCC and Child-Pugh Classification A [12]. The role of these two modalities in the treatment of HCC with TT remains to be investigated by further randomized trials.

Although HCC with TT is not an uncommon condition with an incidence of 44-62.2% [13], the infringement of the tumor to the IVC and RA is a rare presentation indicating a poor prognosis with a median survival of two to five months [14,15]. Up until recently, giving the best supportive care was deemed as the optimal treatment for this patient group [16].

RT did not play a prominent role in the treatment of the HCC due to several limitations. First, as most of these patients have an underlying liver problem, safety is an issue considering that radiation-induced liver injury creates substantial morbidity and even mortality. The liver exhibits a low tolerance to radiation and the tolerance is even less in patients with primary liver cancers [17]. Early on, technical challenges limited the delivery of radiation to the tumor [18]; hence, whole liver irradiation was performed for palliative intent with no additional benefit to survival. The dose that could be safely delivered to the whole liver was ineffective to achieve tumor control [19]; however, with recent advances in CT visualization and treatment delivery techniques, RT was deemed favorable not only for palliative intent but also for therapeutic intent.

In recent years, SBRT has emerged as a game-changer in RT. It permits the delivery of high doses to small volumes. A recent meta-analysis revealed that SBRT provides similar overall survival rates as well as decreased toxicity compared to conventional RT techniques [20]. Because most of these patients have an underlying liver problem, safety is an issue considering that radiation-induced liver injury creates substantial morbidity and even mortality. Given this, adaptive RT should be considered with daily imaging so that tumor coverage and normal tissue sparing would be ensured with tumor response to the treatment as well as daily variations of the patients’ anatomy. A meta-analysis in 2015 evaluated eight studies treating HCC involving the IVC/RA with external beam radiotherapy and documented pooled one- and two-year survival rate of 53.6% and 34.4% without significant heterogeneity between the studies [21]. Only one of them was SBRT. These results are comparable to those obtained with TACE. Similarly, a retrospective analysis of 19 HCC patients with IVC thrombus treated with conventional RT or SBRT showed evidence that RT is feasible and safe with acceptable toxicity in this patient group [22]. SBRT was found to be non-inferior to IMRT regarding overall survival, progression-free survival, and local control with the additional advantage of decreased overall treatment duration [23]. Xi et al. retrospectively evaluated 41 HCC patients with TT in either the portal vein or IVC treated with SBRT. Overall, 36.6% of the patients reached complete response with no grade 4-5 toxicity in any of the patients. They reported that SBRT was safe and effective and that more prospective trials were needed [24]. A subsequent study compared SBRT and three-dimensional conformal radiotherapy for the same patient group and analyses revealed that SBRT results in a higher biologically effective dose than conventional RT, and the local control rates with SBRT are promising in a way that SBRT could be the standard of treatment for this patient group in the future [25].

SBRT requires high precision, accuracy, and reproducibility as it delivers high doses in fewer fractions compared with conventional RT. Even though CT-based approaches targeted the tumor by inhibiting the respiratory motion, respiratory tracking, accounting for the tumor motion by creating a larger safety margin, and tracking marker, interfraction reproducibility was found to be low [26,27]. In addition, the low visibility of liver tumors on X-ray imaging is another limitation for tumor tracking and target precision. Another challenge in liver RT is the nearby tissues that are vulnerable to radiation-induced damage such as luminal gastrointestinal organs and bile ducts. As mentioned above, healthy liver tissue should also be avoided from irradiation at high doses.

MRgRT unites the linear accelerator device with MRI images, and therefore, benefits from the high soft-tissue contrast of MRI. Although MRI-CT fusions have been used for many years, MRgRT enables the acquisition of real-time images during the course of treatment so that irradiation can be performed when the PTV is within the desired target region. MRgRT also allows the user to apply daily adaptive plans with the image of the current fraction. The online system permits the corrections of interfraction as well as intrafractional variations. Other benefits include avoidance of CT radiation dose.

MRgRT has the potential to improve SBRT in HCC patients. The only available retrospective study available in the literature reported the result of 12 patients with HCC and TT treated with MRgRT. Either 50 Gy in 10 fractions or 36-50 Gy in four to five fractions was used. Overall, 83.3% of patients showed an objective response. The study concluded that MRgRT is effective and feasible in HCC with TT in the main trunk or first branch of the portal vein [28].

## Conclusions

Here, to our knowledge, we present the first documented case of HCC with RA extension treated with MRgRT. The tumor was not amenable to surgery nor suitable for other alternative methods due to basal poor prognostic factors. MRgRT allowed us daily planning and tumor tracking with no compromise from our PTV coverage. The change of the tumor location during the treatment was no question for us as the tracking was done online during the whole session under continuous online cine-MRI. No dose violation was noted during any of the fractions which can be attributed to two reasons: (1) our PTV margin was 3 mm which is a margin that cannot be safely given without online continuous tumor tracking, and (2) a new plan was performed before each fraction and any dose violation was intervened before the fraction. The patient responded well to the treatment with no greater than grade 3 side effects and with evidence of complete biochemical response with AFP levels.

MRgRT is a novel approach in RT that leads to tumor tracking and adaptive planning in each fraction resulting in good OAR preservation without comprimising PTV coverage. MRgRT can be safely performed in HCC patients with TT and even RA extension and can be a bridging therapy for orthotopic liver transplantation in this patient group.
